# The biological relevance of potentially toxic metals in freshwater fish

**DOI:** 10.3389/fphys.2025.1609555

**Published:** 2025-09-18

**Authors:** Anton Kovacik, Marek Helczman, Marian Tomka, Tomas Jambor, Eva Kovacikova, Julius Arvay

**Affiliations:** ^1^ Institute of Applied Biology, Faculty of Biotechnology and Food Sciences, Slovak University of Agriculture in Nitra, Nitra, Slovakia; ^2^ Institute of Biotechnology, Faculty of Biotechnology and Food Sciences, Slovak University of Agriculture in Nitra, Nitra, Slovakia; ^3^ Institute of Nutrition and Genomics, Faculty of Agrobiology and Food Resources, Slovak University of Agriculture in Nitra, Nitra, Slovakia; ^4^ Institute of Food Sciences, Faculty of Biotechnology and Food Sciences, Slovak University of Agriculture in Nitra, Nitra, Slovakia

**Keywords:** trace elements, potential toxicity, health, ecotoxicology, fish, biomarker

## Abstract

Trace elements are essential for a number of physiological functions including oxygen transfer, enzymatic reactions and antioxidant protection of the animal organism. Elevated concentrations outside the physiological optimum, on the other hand, can cause undesirable health complications, disrupt metabolic pathways, reproductive capacity, or oxidative balance. The negative anthropogenic impacts on the environment are alarming and the impacts on the aquatic environment have been increasing disproportionately in recent years. Against this background, all potential threats to biota need to be explained and better understood, the possible risks need to be better informed and understood, and a balance needs to be struck between the fundamental nature and the harmful effects of these metals. This mini-review examines the roles of potentially toxic metals including cobalt (Co), copper (Cu), iron (Fe), manganese (Mn), molybdenum (Mo) and zinc (Zn) in fish physiology. This document also elucidates the mechanisms underlying the assessment of regulatory processes, the potential negative consequences of overexposure, the interactions of these metals on fish health, and in the environmental context.

## Graphical Abstract

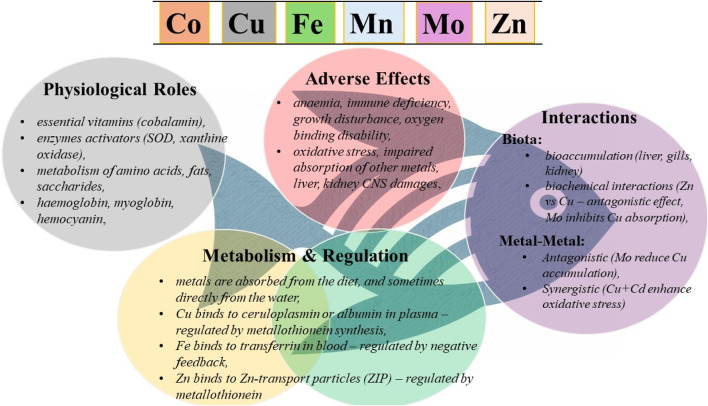

## 1 Introduction

Aquatic environment is complex system, however, human activities, such as industrial discharge, agricultural runoff, and urban wastewater, are increasingly disrupting this delicate balance. This leads to an accumulation of many types of environmental pollutants in the water and sediment (Edo et al., 2024; Khallaf et al., 2018; Kolarova and Napiórkowski, 2021; Kumar et al., 2010). One group of these pollutants are metals, or trace elements. Heavy metals, or potentially toxic elements, are fairly well studied (Dash and Kalamdhad, 2021; Deb and Fukushima, 1999; Kumar et al., 2010). On the other hand, we have the group of potentially toxic metals. These elements (e.g., cobalt, Co, copper, Cu, iron, Fe, manganese, Mn, molybdenum, Mo, or zinc, Zn) are essential for many basic physiological functions of organisms, such as oxygen transport, enzymatic reactions, and the maintenance of antioxidant defence mechanisms (Aliko et al., 2018; Inomata, 2024; Pouil et al., 2016; Wang et al., 2024). They also play a critical role in maintaining the physiological health of fish and are involved in cellular processes and metabolic pathways (Bagheri et al., 2024; Lall, 2003; Zhao et al., 2014). The presence of potentially toxic metals alone does not determine their effects on aquatic animals; the concentration levels and types of interactions they experience in an ecological context also play a role. While some metals may be necessary in small amounts, excessive accumulation above a certain threshold can have adverse toxicological effects (Bury et al., 2003; Chen et al., 2024; Clearwater et al., 2002; Naz et al., 2023). The situation is further complicated by the many interactions between different metals, which may be antagonistic or synergistic in their activities. Elevated metal concentrations can have cascading consequences, including bioaccumulation, oxidative stress, reproductive dysfunction, and impaired biochemical and/or immune systems in fish (de Oliveira et al., 2018; Helczman et al., 2024; Kovacik et al., 2023; Passos et al., 2022; Kovacik et al., 2019). One metal may affect the toxicity or uptake of another metal, creating a dynamic system whose effects on fish populations are unpredictable.

This mini-review aims to summarize the current knowledge about the effects of potentially toxic metals on fish health, focusing on the following points.

• Physiological roles of potentially toxic metals in fish
•Mechanisms of absorption and regulation
•Possible adverse effects of overexposure
•Interactions of these metals the aquatic environment
•Implications for ecological risk assessment and management of aquatic ecosystems

By examining these variables, we can provide a comprehensive and up-to-date summary of recent research on the function of potentially toxic metals in fish health-a broader and more integrative approach than many previous studies, which have focused primarily on contamination levels or toxicological effects. We also aim to identify areas of current ignorance and suggest possible directions for future research based on data from multiple studies. Previous reviews have largely focused on heavy metals as toxic pollutants affecting fish physiology and on the human health risks associated with metal contamination in fish tissues. The emphasis of this review on the physiological functions and regulatory mechanisms of potentially toxic metals, together with an assessment of ecological risks, represents a significant and novel contribution to the field.

## 2 Sources of potentially toxic metals in fish

The accumulation of potentially toxic metals such in the aquatic environment and in fish can be attributed to both natural and anthropogenic sources. Understanding these sources is essential to mitigate the risks associated with metal bioaccumulation in fish and to ensure the sustainability of aquatic ecosystems. The reported concentrations of selected metals in freshwater ecosystem and fish are presented in Supplementary Material (Supplementary Table S1).

### 2.1 Natural sources

The main natural sources of metals in the aquatic environment include geological weathering of rocks, erosion and biological processes in ecosystems. These metals subsequently reach the water and sediments, from where they can naturally, through various forms of transport, enter the body of fish and accumulate there. Fe and Mn are widely distributed elements in the earth’s crust (Jia et al., 2018; Kumar et al., 2010). They are naturally and readily released into aquatic ecosystems by weathering of rocks and soil erosion. They are found mainly in sediments, along with Zn and Cu. Fish may accumulate metals through their diet and interactions with the environment. Some fish species feed on sediments and the organisms residing in them, which facilitates the transfer of metals such as Fe and Mn from sediments into the fish body (Campos et al., 2018; Rajkowska and Protasowicki, 2013). These metals also accumulate in phytoplankton and zooplankton from where they are further transferred to higher trophic levels, including fish (Dash and Kalamdhad, 2021; Kumar et al., 2010). Oxygen levels in water due to seasonal changes can affect the availability of metals in the aquatic environment. Under low O_2_ conditions, there is increased mobility of Fe and Mn in sediments, leading to higher bioaccumulation in fish (Boota et al., 2024).

### 2.2 Anthropogenic sources

The main anthropogenic sources of metals in the aquatic ecosystem include industrial discharges, agricultural runoff, mining activities and wastewater. High levels of metals such as Cu, Zn, and Mn are often found in wastewater from industry. Numerous studies from the Indus River and Nile River channels have confirmed elevated concentrations of these metals in fish due to nearby industrial activities in the area (Boota et al., 2024; Khallaf et al., 2018). The widespread use of fertilizers and pesticides contributes significantly to environmental pollution and the release of metals such as Zn, Cu and Mn into water. Fe, Zn and Cu are often found in high levels in wastewater whether as a result of corrosion of pipelines or processing of various materials in the metallurgical or electro technical industries. Waste and leachate from mining activities is one of the most significant contributors to pollution of watercourses. Metals such as Fe and Mn enter the aquatic ecosystem through mine water discharges. In the Xiang River in China, mining activities have been identified as the primary source of metal contamination, with fish species showing elevated levels of Zn, Fe and Cu (Jia et al., 2018).

## 3 Biological role of potentially toxic metals in freshwater fish

Essential metals play specific and crucial roles in the fish organism. They are involved in the immune response, physiological processes, antioxidant protection, enzymatic activity as well as overall growth and development (Table 1).

**TABLE 1:** Summary of sources, key roles in the organism and negative effects of selected metals on fish health.

Metal	Main sources	Key role in fish organism	Negative impact on fish health in exceeded concentrations	References
Fe	Geological weathering, industrial and mining effluents, pipeline corrosion	Oxygen transport (haemoglobin, myoglobin); Immune system regulation (ROS production); Antioxidant defence (CAT)	Oxidative stress, ↓ antioxidant enzymes (SOD, CAT); Lipid peroxidation; Gut and liver damage; ↑ susceptibility to pathogens	Cano-Viveros et al. (2021), Chen et al. (2024), Gürkan et al. (2021), Luo et al. (2017), Musharraf and Khan (2019), Singh et al. (2019), Zhao et al. (2014)
Cu	Mining, electrical industry, pesticides	Energy production (cytochrome-c oxidase); Immune system activation (cytokines, macrophages); Tissue integrity maintenance (collagen, elastin synthesis)	Anaemia (↓ RBC, HCT, Hb); Lipid peroxidation, ↓ antioxidant enzymes; Inflammation and tissue degeneration (gills, liver, kidneys, brain); DNA damage; LC50 ∼3.4 mg/L	Bagheri et al. (2024), Clearwater et al. (2002), Fırat et al. (2022), Gheorghe et al. (2017), Kumar M et al. (2023), Liao et al. (2023), Naz et al. (2023), Wang et al. (2024), Zhao et al. (2014)
Zn	Fertilizers, galvanized metals, batteries, sewage effluents	Immune response (T-lymphocytes, macrophages); Antioxidant protection (SOD); Growth and tissue development (growth hormone, IGF-1, DNA repair)	Moderate toxicity (LC50 ∼20.8 mg/L); Enzyme disruption (GST, CAT); Oxidative stress → lipid peroxidation and tissue damage	Banni et al. (2011), Clearwater et al. (2002), Dawood et al. (2022), Gheorghe et al. (2017), Kumar et al. (2017), Wang et al. (2020), Xu X. W. et al. (2023), Zhao et al. (2014)
Mn	Metallurgical industry, natural geological weathering	Carbohydrate and lipid metabolism (Mn-SOD); Bone mineralization and connective tissue formation; Reduce inflammation; Increased antioxidant capacity and enzyme activity	Genotoxicity (DNA damage, micronuclei); Enzyme alteration (GST, CAT); Lower acute toxicity vs other metals (LC50 ∼53 mg/L)	Aliko et al. (2018), Bagheri et al. (2024), Gheorghe et al. (2017), Musharraf and Khan (2021), Passos et al. (2022), Xu J. J. et al. (2023)
Co	Fossil fuel combustion, industrial wastewater	Vitamin B_12_ synthesis (energy metabolism, nervous system function); Fatty acid and nucleic acid metabolism; Support for cell division and growth	Oxidative stress, lipid peroxidation, apoptosis, DNA damage; Disrupts reproduction and enzyme metabolism; Alters Ca^2+^ homeostasis (competition)	Bagheri et al. (2024), Banerjee and Ragsdale (2003), Blust (2011), Ghribi et al. (2025), Javed and Usmani (2013), Kubrak et al. (2011), Younus et al. (2020)
Mo	Fertilizers, pesticides, industrial sewage, chemical waste	Sulphur metabolism and detoxification (enzyme cofactor); Amino acid metabolism	Limited data; Potential oxidative stress and enzyme dysfunction	Aubakirova et al. (2023), Regoli et al., 2012; Reid (2002), Ricketts et al. (2015), Satkanov et al. (2025)

Co is one of the less studied trace elements for fish health but plays several known functions. It is a key component of vitamin B_12_, which is essential for energy metabolism and proper nervous system function. Acts as a cofactor for enzymes involved in fatty acid synthesis and nucleic acid metabolism (Banerjee and Ragsdale, 2003; Blust, 2011). Supports cell division and differentiation and is essential for normal growth and development of cyprinid fish *Tor putitora*, especially in the early stages of life (Younus et al., 2020).

Cu is a cofactor of several enzymes such as cytochrome-c oxidase and superoxide dismutase (Cu/Zn-SOD - in the cytosol), which play a role in energy production and antioxidant defence (Clearwater et al., 2002; Wang et al., 2024). It plays a role in cytokine production, and is involved in the activation of immune cells such as macrophages and lymphocytes (Bagheri et al., 2024). It is an essential component for the synthesis of elastin and collagen, critical for tissue integrity and wound healing (Wang et al., 2024). Participates in the nervous system health, neurotransmitter synthesis and synaptic function in stressed zebrafish (Green et al., 2024).

Fe is a vital element for various biological processes in fish, including oxygen transport, enzyme function, and immune response. As a key component of haemoglobin and myoglobin, Fe is essential for oxygen transport and energy metabolism in fish (de Oliveira et al., 2018). It is involved in the regulation of immune cells and the production of reactive oxygen species (ROS) required for defence against pathogens (de Oliveira et al., 2018; Musharraf and Khan, 2019). It is part of the antioxidant capacity, as a component of catalase (CAT), an iron-containing haemoprotein that breaks down hydrogen peroxide (H_2_O_2_) into water and oxygen, thereby protecting cells from oxidative damage (de Oliveira et al., 2018; Luo et al., 2017). Dietary Fe improves growth performance, haematology and gut health of fish *Labeo rohita* (Musharraf and Khan, 2019), especially when provided in a nanoform that increases bioavailability.

Mn is a cofactor of enzymes involved in carbohydrate metabolism, lipid metabolism. Plays role in antioxidant protection as part of the superoxide dismutase (Mn-SOD) (de Oliveira et al., 2018; Musharraf and Khan, 2021). Mn could impact bone mineralization and connective tissue protein synthesis in salmonids (Baeverfjord et al., 2019). It is important for the growth performance, fatty acid uptake or triglycerides deposition. Mn also could reduce inflammation, increased antioxidant capacity and enzyme activity in *Carassiud auratus* or *Pelteobagrus fulvidraco* (Aliko et al., 2018; Xu J. J. et al., 2023).

Mo is a cofactor of enzymes involved in sulphur metabolism and detoxification processes (Inomata, 2024; Lall, 2003). It is essential for the metabolism of amino acids and other biomolecules (Lall, 2003). Recent studies suggest a significant biological relevance, namely in the context of nitric oxide production from nitrate and nitrite by increasing the activity of the molybdoenzymes xanthine oxidase (XO) and aldehyde oxidase (AO) in the liver in *Silurus glanis* (Aubakirova et al., 2023). Another study provides important insights into the ability of a Mo cofactor (Mo-co) isolated from the protein fraction of fish liver extract to restore NADPH-nitrate reductase (NADPH-NR) activity (Satkanov et al., 2025).

Zn is one of the best-studied elements in fish nutrition and physiology. It is critical for the activation and function of T-lymphocytes and macrophages and is involved in the production of antibodies. Participates in the regulation of immune response-related gene expression in *Oreochromis niloticus* (El-Sayed et al., 2023). Acts as a cofactor for antioxidant enzymes such as SOD, and helps protect cells from oxidative damage in zebrafish or *Pangasius hypophthalmus* (Banni et al., 2011; Kumar et al., 2017). Regulates endocrine signalling pathways (growth hormone and IGF-1) that influence body growth, tissue differentiation and bone ossification. It is part of enzymes that regulate important biological functions including DNA repair, RNA formation and amino acid metabolism (Dawood et al., 2022; Wang et al., 2020). It is required for the activity of enzymes involved in DNA replication, and its absence causes growth inhibition and abnormalities (Clearwater et al., 2002), Specifically, increased growth performance, improved antioxidant capacity and inflammatory responses were confirmed in *P. fulvidraco* (Xu X. W. et al., 2023).

## 4 Key pathways and uptake mechanisms in the accumulation of metals in fish

**FIGURE 1:**
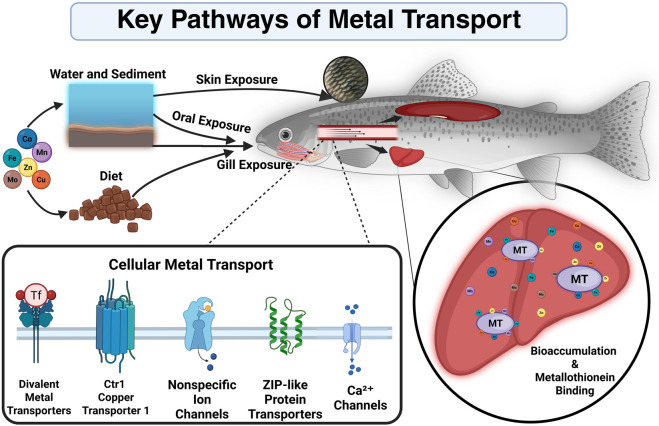
Key exposure routes, cellular uptake mechanisms, and intracellular interactions of metals in fish (Created in BioRender. Helczman, 2025).

The accumulation of potentially toxic metals in freshwater fish involves a complex interplay of uptake pathways and biological mechanisms (Figure 1). The main uptake pathways include uptake through the food and water, and indirect uptake through the skin and mucous membranes.

Gills are considered to be the primary source of uptake of metals from the aquatic environment into the fish. Gills play a critical role in ion regulation and gas exchange, and are directly and continuously exposed (Deb and Fukushima, 1999; Zia and McDonald, 1994). Chloride cells found in the gills of fish are specialized cells that are involved in ion transport, especially Ca^2+^. They may also play a role in the uptake of some metals, especially when present as divalent cations (Zia and McDonald, 1994).

Through ionic bonds or covalent bonds with cytosolic compounds, these metals can bind to the gill surface. The affinity is different in this process, with Cu showing a higher affinity compared to Fe or Zn.

Fish accumulate high levels of metals through ingestion of food or sediment (Bury et al., 2003; Pouil et al., 2016). Assimilatory efficiency plays an important role in the transport of metals across the intestinal wall, which varies greatly depending on the specific metal. For example, Zn and Cu have a higher assimilative efficiency than Co or Mo, which may lead to their more efficient absorption in the gut and consequently higher accumulation in the body of *Oncorhynchus mykiss* (Ojo and Wood, 2007).

Particularly, in the presence of high concentrations of metals in the aquatic environment, absorption of metals also occurs through the skin and mucous membranes of fish (Deb and Fukushima, 1999). This process can be influenced by factors such as the chemical form of the metal, water temperature, pH and the physiological state of the fish.

Metals enter fish cells through ion channels and transporters. While Cu and Zn can enter cells through Ca^2+^ channels, whereas Fe uptake is mediated by specific iron transporters such as DMT1 (divalent metal transporter 1) or transferrin receptors (Bury et al., 2003; Deb and Fukushima, 1999). Metals can interact with each other in their absorption and biological effects. For instance, excess zinc (Zn) may reduce copper (Cu) absorption by stimulating the production of metallothionein’s, which bind copper and prevent its absorption. Similarly, iron (Fe) can increase the toxicity of manganese (Mn) by promoting its accumulation in the body, especially in the brain, which can lead to neurotoxic effects (Clearwater et al., 2002; de Oliveira et al., 2018).

Specific proteins and metallothionein (MT) play the most important role in maintaining metal homeostasis and detoxification of the body (Deb and Fukushima, 1999). MT´s are cysteine-rich proteins that bind metals via thiol groups. They are involved in the detoxification of metals such as Cu and Zn. Metals are bound by these mechanisms after entering the fish body and subsequently either stored or excreted for proper regulation.

The bioavailability of metals is influenced by the chemical parameters of the water (pH, hardness and the presence of ligands). The toxicity of some metals such as Cu is influenced by the formation of hydroxide complexes in circumneutral waters (Liao et al., 2023).

The gills, as an organ constantly exposed to direct contact with water and food, and the liver, as a major detoxification organ, are among the primary tissues of metal accumulation. Numerous studies have demonstrated high accumulation of metals such as Fe, Cu and Zn in the liver (Javed and Usmani, 2013; Jia et al., 2017; Spanopoulos-Zarco et al., 2017). Kidney is also one of the organs with high accumulation, as it plays an important role in excretion of excess metals. However, the efficiency of excretion varies depending on both the metal and the degree of accumulation (Deb and Fukushima, 1999; Javed and Usmani, 2013).

## 5 Potential toxicity of metals in fish

Although the monitored elements are biogenic and play various roles in the fish organism, maintaining their optimal levels is crucial for the health of the organism. Their deficiency or excessive accumulation can lead to physiological and biochemical disturbances, that may have toxic effects on cellular and systemic processes. In general, excessive levels of metals can cause oxidative stress, DNA damage and disruption of enzyme activity, leading to impaired growth, reproduction and survival (de Oliveira et al., 2018; Naz et al., 2023). Also, metal speciation in water, i.e. the different chemical forms in which these metals occur in the environment, directly affects their effectiveness and uptake/absorption into the organism. Free metal ions are more bioavailable, and fish tissues/mucous membranes absorb them much more easily. On the other hand, metals in organic or inorganic compounds are less bioavailable (De Paiva Magalhães et al., 2015; Millero, 2001). Metal speciation, and thus of course bioavailability, is also affected by environmental changes (e.g. pH, salinity, hardness, and oxygen or carbon content) (Dahlberg et al., 2025; Pierrot and Millero, 2017; Rouleau et al., 1996); for example, significant changes in pH (either decrease or increase) combined with factors such as water hardness and the presence of different metals, influence metal speciation by maintaining metals in their free ionic forms, which can increase absorption and associated toxicity in fish (De Paiva Magalhães et al., 2015; Namieśnik and Rabajczyk, 2010).

Excessive dietary Fe exposure can lead to oxidative stress, decreased activity of antioxidant enzymes (e.g., superoxide dismutase, catalase), and increased lipid peroxidation (Cano-Viveros et al., 2021; Gürkan et al., 2021), specifically in the liver of *Prochilodus lineatus* (de Oliveira et al., 2018). This suppression of immunity makes fish more susceptible to pathogens such as *Aeromonas hydrophila* in *Micropterus salmoides* (Chen et al., 2024). High Fe levels can also damage intestinal, gills and liver tissues in *Labeo rohita* and alter the composition and function of the gut microbiota (Chen et al., 2024; Singh et al., 2019), specifically in *M. salmoides*.

Exposure to copper sulphate or copper oxide nanoparticles (CuO-NPs) decreases erythrocyte count, haematocrit, and haemoglobin levels, indicating anaemia. Cu exposure increases lipid peroxidation and decreases the activity of antioxidant enzymes (e.g., SOD, CAT). It also causes inflammation and degenerative changes in gills, liver, kidney and brain tissues (Fırat et al., 2022; Naz et al., 2023), and DNA damage (Fırat et al., 2022; Kumar M et al., 2023; Passos et al., 2022) in *L. rohita*, *Oreochromis niloticus*, and/or *Channa punctata*.

The 96-h LC_50_ for Zn in freshwater fish (e.g., *Cyprinus carpio*) is approximately 20.8 mg/L, indicating moderate toxicity (Gheorghe et al., 2017). Zn toxicity affects the activity of enzymes involved in detoxification, including increased expression of glutathione-S-transferase (GST) and catalase (CAT) (Passos et al., 2022). This response can lead to oxidative stress, which causes lipid peroxidation and subsequent tissue damage.

Mn in combination with Fe induces genotoxic effects including DNA damage and micronucleus formation in *O. niloticus* (Passos et al., 2022). It also alters the activity of thyroid function, hepatic, GST and CAT enzymes (Hoseini et al., 2014; Kumar N et al., 2023). The LC_50_ for Mn in freshwater fish *C. carpio* is relatively high (>53 mg/L), indicating lower acute toxicity compared to other metals (Gheorghe et al., 2017).

Co toxicity can induce oxidative stress leading to lipid peroxidation, cell apoptosis and DNA damage in fish tissues (e.g. in *Carassius auratus*) (Blust, 2011; Kubrak et al., 2011). High levels interfere with fish reproductive health, enzyme metabolism and calcium balance due to competition (Bagheri et al., 2024; Ghribi et al., 2025).

In the case of Mo, there is insufficient information on its effects on fish health in excessive accumulation, but it is suggested that oxidative stress and dysfunction of some enzymes may occur. The 96-h LC50 values range from >50 to >10,000 mg L^-1^, with these values varying widely depending on the species (Reid, 2002; Ricketts et al., 2015). Exceeding toxic levels can lead to increased Mo accumulation in tissues and potential toxicity (Regoli et al., 2012). Despite its low toxicity, physiological changes have been observed even at sublethal concentrations, including accelerated breathing, loss of balance after physical exertion, and tissue damage such as fused gill lamellae and haemorrhages in the digestive tract (Reid, 2002; Ricketts et al., 2015). Interesting are also the interactions of Mo in nanoforms, e.g. MoS_2_, which can increase the toxicity of other metals, such as antimony, through oxidative stress and disruption of fatty acid metabolism in algae (Zou et al., 2024).

## 6 Future research and recommendations

Research on trace metals in fish is a key area for understanding their dual impact as essential elements and potential toxicants. The first priority should be to develop advanced, more sensitive and cost-effective methods for realistic monitoring of metal concentrations in the aquatic ecosystem. The use of modern techniques and artificial intelligence appears to be a promising step. Advanced AI algorithms can process large monitoring datasets, identify patterns of biomarker changes based on metal concentrations and environmental conditions, and generate predictive models for the early detection of risk and real-time prognosis of toxic effects. It is also important to identify new biomarkers and establish early warning systems to detect exposure and toxicity. Promising new biomarkers may include microRNAs that regulate stress pathways, as well as transcriptomic and metabolomic profiles that are sensitive to microelements. Proteomic analyses can identify proteins that enable the detection of sublethal toxicity before traditional oxidative stress symptoms appear. Furthermore, it is important to investigate the interactive effects of metals, which may have synergistic but also antagonistic effects on fish physiology. Developing models to predict these combined effects under different conditions would provide a deeper understanding of the complex interactions. Given changes in climatic conditions (temperature change, ocean acidification, shifts in hydrological cycles), it is essential to investigate the influence of these factors on the dynamics of metal toxicity.

## 7 Conclusion

This review discusses the intricate relations between potentially toxic metals in freshwater fish and their significance to fish physiology along with the possible detrimental effects of their accumulation. The interaction of their beneficial and adverse effects emphasizes the necessity of integrated management strategies in aquatic environments.

The role of cobalt, copper, iron, zinc, molybdenum and manganese in fish physiological processes and the precarious balance between natural and man-made sources of metals in aquatic systems are considerations of fundamental importance. The potential toxicity of even the essential elements, the determinants of metal toxicity and bioavailability, and the long-term ecological implications of metal accumulation in fish are also among the major conclusions of this study.

Future research should be directed predominantly at enhancing monitoring and early warning systems, examining the impacts of metal mixtures as well as their interactions with environmental stressors, developing novel solutions to pollution remediation, exploring the genetic and epigenetic impacts of chronic metal exposure, and refining risk model prediction associated with human health.

In conclusion, the regulation of potentially toxic metals in freshwater systems requires an integrative interdisciplinary strategy. Enabling a clearer understanding of such complex interactions will allow us to more effectively develop methodologies aimed at protecting the aquatic ecosystem, healthy fish populations, and protecting human health in an environment of increasing environmental pressures. The findings of this review can be translated into environmental monitoring and regulatory frameworks by identifying the most sensitive biomarkers of metal exposure, integrating AI-driven predictive models to detect early signs of toxicity, and informing evidence-based thresholds for water quality standards that account for sublethal and synergistic effects of metals.
