# Correction: The biological relevance of potentially toxic metals in freshwater fish

**DOI:** 10.3389/fphys.2025.1717005

**Published:** 2025-10-28

**Authors:** Anton Kovacik, Marek Helczman, Marian Tomka, Tomas Jambor, Eva Kovacikova, Julius Arvay

**Affiliations:** ^1^ Institute of Applied Biology, Faculty of Biotechnology and Food Sciences, Slovak University of Agriculture in Nitra, Nitra, Slovakia; ^2^ Institute of Biotechnology, Faculty of Biotechnology and Food Sciences, Slovak University of Agriculture in Nitra, Nitra, Slovakia; ^3^ Institute of Nutrition and Genomics, Faculty of Agrobiology and Food Resources, Slovak University of Agriculture in Nitra, Nitra, Slovakia; ^4^ Institute of Food Sciences, Faculty of Biotechnology and Food Sciences, Slovak University of Agriculture in Nitra, Nitra, Slovakia

**Keywords:** trace elements, potential toxicity, health, ecotoxicology, fish, biomarker

Authors [**Anton Kovacik** and **Marek Helczman**] were erroneously omitted as equal contributing authors.

The original article has been updated.

