# Supplementary material for: The biological relevance of potentially toxic metals in freshwater fish
Source: Front Physiol. 2025 Sep 18;16:1609555. doi: 10.3389/fphys.2025.1609555 (PMC12488562; doi:10.3389/fphys.2025.1609555)
Supplement: Supplementary file 1 [file Table1.docx]

**Table S1** Reported concentrations of selected metals in freshwater ecosystem and fish.

| **Fish** | **Locality** | **Concentrations in water/sediment (μg/g unless stated otherwise)** | **Concentrations in fish/tissues (μg/g unless stated otherwise)** | **Source of contamination** | **References** |
| --- | --- | --- | --- | --- | --- |
| *Labeo rohita*  *Tor putitora* | River Indus, Pakistan (from 34°03'49.1"N 72°40'05.5"E to 33°58'40.3"N 72°22'58.8"E | Cu: BDL/BDL to 389  Fe: BDL to 18.9/BDL  Mn: BDL/BDL to 501  Zn: BDL to 10.7/BDL to 670 | *L. rohita* (muscle): Cu – 5.19; Fe – 17.38; Mn – 21.02; Zn – 38.18  *T. putitora* (muscle): Cu – 5.30; Fe – 17.65; Mn – 22.03; Zn – 39.01 | NS | (Boota et al., 2024) |
| *Notopterus notopterus*  *Clarias batrachus*  *Channa striata* | Deepor Beel, India | Cu: cca 180 – 780 μg/L/cca 18 – 45 mg/kg  Fe: cca 200 – 980 μg/L/cca 7500 – 8900 mg/kg  Mn: cca 200 – 490 μg/L/cca 320 – 380 mg/kg | *N. notopterus* (muscle, mg/kg d.w.)  Cu – 0.910 – 1.143; Fe – 22.822 – 28.155; Mn – 0.420 – 0.886  *N. notopterus* (liver, mg/kg d.w.)  Cu – 3.486 – 4.513; Fe – 58.093 – 77.664; Mn – 0.816 – 1.483  *N. notopterus* (gill, mg/kg d.w.)  Cu – 2.387 – 3.204; Fe – 40.449 – 44.499; Mn – 0.189 – 0.270  *N. notopterus* (skin, mg/kg d.w.)  Cu – 2.041 – 2.655; Fe – 45.607 – 54.589; Mn – 0.649 – 1.386  *C. batrachus* (muscle, mg/kg d.w.)  Cu – 0.904 – 1.124; Fe – 21.622 – 28.603; Mn – 0.408 – 0.848  *C. batrachus* (liver, mg/kg d.w.)  Cu – 3.451 – 4.405; Fe – 58.706 – 77.358; Mn – 0.793 – 1.413  *C. batrachus* (gill, mg/kg d.w.)  Cu – 2.401 – 3.248; Fe – 39.823 – 49.410; Mn – 0.190 – 0.275  *C. batrachus* (skin, mg/kg d.w.)  Cu – 2.054 – 2.695; Fe – 38.163 – 57.718; Mn – 0.664 – 1.434  *C. striata* (muscle, mg/kg d.w.)  Cu – 0.903 – 1.121; Fe – 21.857 – 28.779; Mn – 0.406 – 0.842  *C. striata* (liver, mg/kg d.w.)  Cu – 3.409 – 4.273; Fe – 50.811 – 74.857; Mn – 0.766 – 1.328  *C. striata* (gill, mg/kg d.w.)  Cu – 2.416 – 3.294; Fe – 42.054 – 45.607; Mn – 0.192 – 0.279  *C. striata* (skin, mg/kg d.w.)  Cu – 2.032 – 2.628; Fe – 44.435 – 53.484; Mn – 0.638 – 1.354 | Solid waste landfill, agriculture, industry | (Dash and Kalamdhad, 2021) |
| *Cyprinus carpio* | Kolinany, Slovakia (48°21’14.6“N 18°13’03.2“E) | NS | Co (serum) – 0.01  Co (ejaculate) – 0.04  Mo (serum) – 0.05  Mo (ejaculate) – 0.014 | Agricultural activity, local wastewater treatment | (Helczman et al., 2024) |
| *Mastacembelus armatus* | Kasimpur, Aligarh, India (27.218°N; 79.378°E | Co – 0.11 mg/L  Cu – 0.86 mg/L  Fe – 8.76 mg/L  Mn – 0.21 mg/L  Zn – 0.3 mg/L | Co (mg/kg d.w.) – gills and kidney – ND; liver – 25.6; muscle – 9.06; integument – 9.06  Cu (mg/kg d.w.) – gills – 199.88; kidney – 175.89; liver – 271.67; muscle – 41.36; integument – 36.27  Fe (mg/kg d.w.) – gills – 799.66; kidney – 649.76; liver – 2601.49; muscle – 213.29; integument – 313.36  Mn (mg/kg d.w.) – gills – 25.36; kidney – ND; liver – 49.96; muscle – 9.03; integument – 13.62  Zn (mg/kg d.w.) – gills – 549.33; kidney – 351.28; liver – 1741.95; muscle – 186.19; integument – 168.11 | Wastewater of Harduaganj Thermal Power Plant | (Javed and Usmani, 2013) |
| *Carassius auratus*  *Squaliobarbus curriculus*  *Pelteobagrus fulvidraco*  *Silurus asotus* | Xiang River, China |  | Muscle (mg/kg dry weight):  *C. auratus*: Co – 0.025; Cu – 3.39; Fe – 42.7; Mn – 2.69; Zn – 53.4  *S. curriculus*: Co – 0.088; Cu – 3.05; Fe – 33.4; Mn – 3.03; Zn – 30.1  *P. fulvidraco*: Co – 0.034; Cu – 1.98; Fe – 25.5; Mn – 2.28; Zn – 26.5  *S. asotus*: Co – 0.026; Cu – 1.52; Fe – 22.4; Mn – 1.37; Zn – 24.4 | Industrial wastewater | (Jia et al., 2018) |
| *Cyprinus carpio*  *Ctenopharyngodon idella* | Kolinany, Slovakia (48°21’14.6“N 18°13’03.2“E) | For *C. idella* study:  Co – BDL/9.231-16.578 mg/kg  Cu – 0-0.096/7.342-78.113 mg/kg  Fe – BDL/17,502-36,084 mg/kg  Mn – BDL/273.8-536.7  Mo – BDL/BDL  Zn – BDL/20.53-352.9 mg/kg | *C. carpio* (blood serum; mg/kg):  Cu – 1 – 2.6 mg/L; Fe – 5 – 52 mg/L; Mn – 0.02 – 0.9 mg/L; Zn – 4.42 – 119.64 mg/L  *C. idella* (blood serum; mg/kg):  Co – 0 – 0.418; Cu – 0 – 2.273; Fe – 1.526 – 12.224; Mn – BDL; Mo – BDL; Zn – 4.650 – 12.269  *C. idella* (blood clot; mg/kg):  Co – 0 – 0.243; Cu – 0.215 – 0.561; Fe – 216.9 – 603.9; Mn – 0 – 0.146; Mo – BDL; Zn – 5.103 – 10.516 | Agricultural activity | (Kovacik et al., 2023, 2019) |
| *Esox lucius*  *Abramis brama* | Lake Ińsko and Lake Wisola, Poland | Lake Ińsko (μg/l; μg/g dry weight):  Fe – 21.8/21,840  Mn – 8.5/256  Zn – 10.0/138.3  Cu – 0.66/17.5  Lake Wisola (μg/l; μg/g dry weight):  Fe – 29.7/13,774  Mn – 25.1/189  Zn – 17.1/149.6  Cu – 0.51/19.5 | E. lucius (muscle; μg/g wet weight):  Fe – 1.5; 1.3; Mn – 0.4; 0.2; Zn – 3.2; 3.0; Cu – 0.18; 0.24  E. lucius (gills; μg/g wet weight):  Fe – 24.8; 1.3; Mn – 0.4; 0.2; Zn – 3.2; 3.0; Cu – 0.18; 0.24  E. lucius (liver; μg/g wet weight):  Fe – 55.9; 29.7; Mn – 0.7; 0.7; Zn – 42.5; 29.1; Cu – 2.64; 1.87  E. lucius (kidney; μg/g wet weight):  Fe – 53.1; 45.4; Mn – 0.3; 0.3; Zn – 70.5; 64.4; Cu – 0.51; 0.60  E. lucius (DT; μg/g wet weight):  Fe – 8.6; 6.5; Mn – 0.8; 0.7; Zn – 559.0; 448.3; Cu – 0.52; 0;51  E. lucius (gonads; μg/g wet weight):  Fe – 9.3; 5.5; Mn – 1.3; 1.5; Zn – 34.7; 43.3; Cu – 0.49; 0.33  E. lucius (spleen; μg/g wet weight):  Fe – 87.7; 93.4; Mn – 0.2; 0.3; Zn – 26.5; 21.2; Cu – 0.32; 0.33  E. lucius (skin; μg/g wet weight):  Fe – 3.9; 3.5; Mn – 5.7; 6.3; Zn – 115.2; 102.7; Cu – 0.48; 0.50  E. lucius (DTC; μg/g wet weight):  Fe – 15.8; 8.0; Mn – 2.2; 1.5; Zn – 216.8; 154.8; Cu – 0.92; 0.52  *A. brama* (muscle; μg/g wet weight):  Fe – 1.5; 1.3; Mn – 0.4; 0.2; Zn – 3.2; 3.0; Cu – 0.18; 0.24  *A. brama* (gills; μg/g wet weight):  Fe – 24.8; 47.5; Mn – 8.8; 9.4; Zn – 12.3; 19.8; Cu – 0.49; 0.59  *A. brama* (liver; μg/g wet weight):  Fe – 54.9; 71.3; Mn – 0.8; 0.8; Zn – 12.3; 12.8; Cu – 4.07; 4.61  *A. brama* (kidney; μg/g wet weight):  Fe – 72.1; 68.3; Mn – 0.6; 0.6; Zn – 8.2; 7.5; Cu – 0.53; 0.49  *A. brama* (DT; μg/g wet weight):  Fe – 15.3; 9.4; Mn – 1.4; 0.7; Zn – 13.1; 10.6; Cu – 0.80; 0.57  *A. brama* (gonads; μg/g wet weight):  Fe – 7.1; 8.4; Mn – 2.1; 2.2; Zn – 26.7; 29.3; Cu – 0.94; 0.85  *A. brama* (spleen; μg/g wet weight):  Fe – 106.1; 152.0; Mn – 0.8; 0.5; Zn – 10.9; 14.3; Cu – 0.98; 0.94  *A. brama* (skin; μg/g wet weight):  Fe – 4.2; 3.1; Mn – 8.0; 8.4; Zn – 36.7; 46.8; Cu – 0.65; 0.44  *A. brama* (DTC; μg/g wet weight):  Fe – 70.8; 53.1; Mn – 4.0; 2.9; Zn – 13.1; 11.5; Cu – 1.24; 0.86 | Lake Ińsko: holiday centers  Leke Wisola: municipal sewage treatment plant | (Rajkowska and Protasowicki, 2013) |

BDL – Below detection limit; NS – not stated; d.w. – dry weight; ND – not detected; DT – digestive tract; DTC – content of digestive tract.
